# Colour contrast sensitivity in eyes at high risk of neovascular age-related macular degeneration

**DOI:** 10.1177/1120672119866386

**Published:** 2019-08-14

**Authors:** Antonio Calcagni, Olivia Howells, Frank Eperjesi, Hannah Bartlett, Alastair KO Denniston, Jonathan M Gibson, Christopher R Hogg, Timothy D Matthews

**Affiliations:** 1 The School of Life & Health Sciences, Aston University, Birmingham, UK; 2 University Hospitals Birmingham NHS Foundation Trust, Birmingham, UK; 3 Moorfields Eye Hospital NHS Foundation Trust, London, UK; 4 Medical Innovation Development Research Unit (MIDRU), Heart of England NHS Foundation Trust, Birmingham, UK; 5 Academic Unit of Ophthalmology, University of Birmingham, Birmingham, UK

**Keywords:** Age-related macular degeneration, colour vision, contrast sensitivity, psychophysics, ChromaTest

## Abstract

**Purpose:**

To generate the first published reference database of colour contrast sensitivity in eyes at high risk of neovascular age-related macular degeneration and to explore this important feature in quality of vision.

**Background:**

Quality of vision depends on many factors. Changes in chromatic contrast sensitivity remain largely unexplored in eyes at high risk of neovascular age-related macular degeneration; they may however not only be relevant for quality of life but also an early indicator of the onset of the disease, so it is important to have a means to evaluate any variation in colour contrast sensitivity, especially in view of the likely increase in neovascular age-related macular degeneration as the population ages.

**Methods:**

This prospective longitudinal study evaluated colour contrast sensitivity along the protan and tritan colour axes in 145 eyes at high risk of neovascular age-related macular degeneration.

**Results:**

Colour contrast sensitivity showed statistically significant correlations with age and visual acuity, but not gender nor laterality (i.e. whether the right or left eye was being tested). There was significant variability among individuals, especially for the tritan axis, with some subjects well within normal limits for age and others with very poor colour contrast sensitivity.

**Conclusion:**

This study has generated the first published colour contrast sensitivity reference database for eyes at high risk of neovascular age-related macular degeneration. It has also shown a high inter-individual variability of colour contrast sensitivity in eyes at high risk of neovascular age-related macular degeneration, but the significance of this is unclear. Further work is required to establish if eyes with high colour contrast sensitivity thresholds (i.e. poor colour vision) have a higher risk of developing neovascular age-related macular degeneration over time, and this is the subject of ongoing work.

## Introduction

Age-related macular degeneration (AMD) is the leading cause of irreversible sight impairment in people older than 50 years in Western society.^1–3^ As the population ages, AMD is likely to become a much more significant issue,^2^ with estimates suggesting numbers will more than double by 2050.^4^ It is a complex multifactorial disease, with genetic as well as environmental factors involved in its pathogenesis.^5–7^ Previous studies^8^ have noted function in eyes at high risk of neovascular age-related macular degeneration (nAMD) is frequently impaired, and it has been suggested^9–13^ that retinal changes in high-risk eyes determine abnormal function in the adjacent photoreceptors/inner retina, with some cone populations affected more than others.^12–14^ Normal colour vision involves comparison of signals generated in short-wavelength (S), medium-wavelength (M) and long-wavelength (L) sensitive cones. Under optimal conditions, trichromats require, at threshold, a smaller contrast change to detect red–green colour differences than yellow–blue colour differences, possibly related to the S-cones being much less numerous than M- and L-cones, especially in the foveal region. In view of the high sensitivity for colour detection and the complexity of chromatic processing, colour assessment is particularly suitable for detecting early stages of retinal disease. To date, however, whilst several vision attributes have been studied in AMD, changes in chromatic sensitivity remain largely unexplored and a colour vision database of eyes at high risk of nAMD, against which tested eyes can be compared, has not been published.

The investigators evaluated colour contrast sensitivity (CCS) as measured with the ChromaTest^8^ in eyes at high risk of developing nAMD, in an attempt to better understand cone photoreceptor function in this context and provide a reference database for any future studies aiming to evaluate colour vision in eyes at high risk of nAMD.

## Methods

Participants were recruited across three UK sites from January 2015 to July 2016, and CCS was measured in the fellow unaffected eye of individuals with unilateral nAMD, which has a 12%–15% yearly risk of developing the pathology.^15^

### Study design

In this prospective longitudinal study, subjects aged 50 years or over with unilateral (treated or inactive) nAMD were eligible to take part. Mild dry age-related changes in the fellow tested eye were not considered a reason for ineligibility for recruitment to this study. Exclusion criteria were individuals with significant media opacities (defined as the impossibility to adequately assess the retina with a 90-dioptre lens on slit lamp biomicroscopy), visual acuity worse than 0.2 LogMAR, pathology that could affect CCS, inherited colour vision deficiencies, high refractive error (established at 7 dioptres spherical equivalent) and individuals not fluent in the English language or unable to give informed consent.

As this study set out to generate a reference database of high-risk eyes free of active nAMD/retinal disease, only patients who did not develop a choroidal neovascular membrane or other significant macular pathology in the tested eye for at least 6 months following CCS assessment were included in the analysis. If the protan large letter CCS test was above 25%, suggesting an undiagnosed congenital colour vision impairment, results were not included in the analysis.

Informed written consent was obtained from all eligible participants who agreed to take part, all applicable institutional and governmental regulations were followed and the study adhered to the tenets of the Declaration of Helsinki.

### Assessments

Monocular visual acuity was assessed and CCS measured only in the eye not affected by nAMD. The CCS assessment has been amply described previously.^8^ In brief, it is divided into four individual sub-tests: protan small letter test, tritan small letter test, protan large letter test and tritan large letter test. Protan tests assess the red–green colour axis. Tritan tests assess the blue–yellow colour axis. All ChromaTest assessments present a letter in the centre of the monitor, with small letter tests subtending 1.5° of visual angle and large letter tests subtending 4° of visual angle. Colour contrast was defined as 0% when the letter had the same hue as the background and 100% when the difference in colour between the letter and the background was at its maximum achievable by the monitor; CCS was determined as the minimum contrast required to identify the letter correctly.

### Data analysis and power calculation

Microsoft Excel and IBM SPSS were used for data analysis. Measured CCS values were used to create a reference database for eyes at high risk of nAMD and descriptive statistical parameters of CCS in the unaffected eye of patients with unilateral nAMD.

The CCS values were also analysed against visual acuity, age and gender, laterality (right or left eye) and site of assessment.

## Results

**Figure 1.**
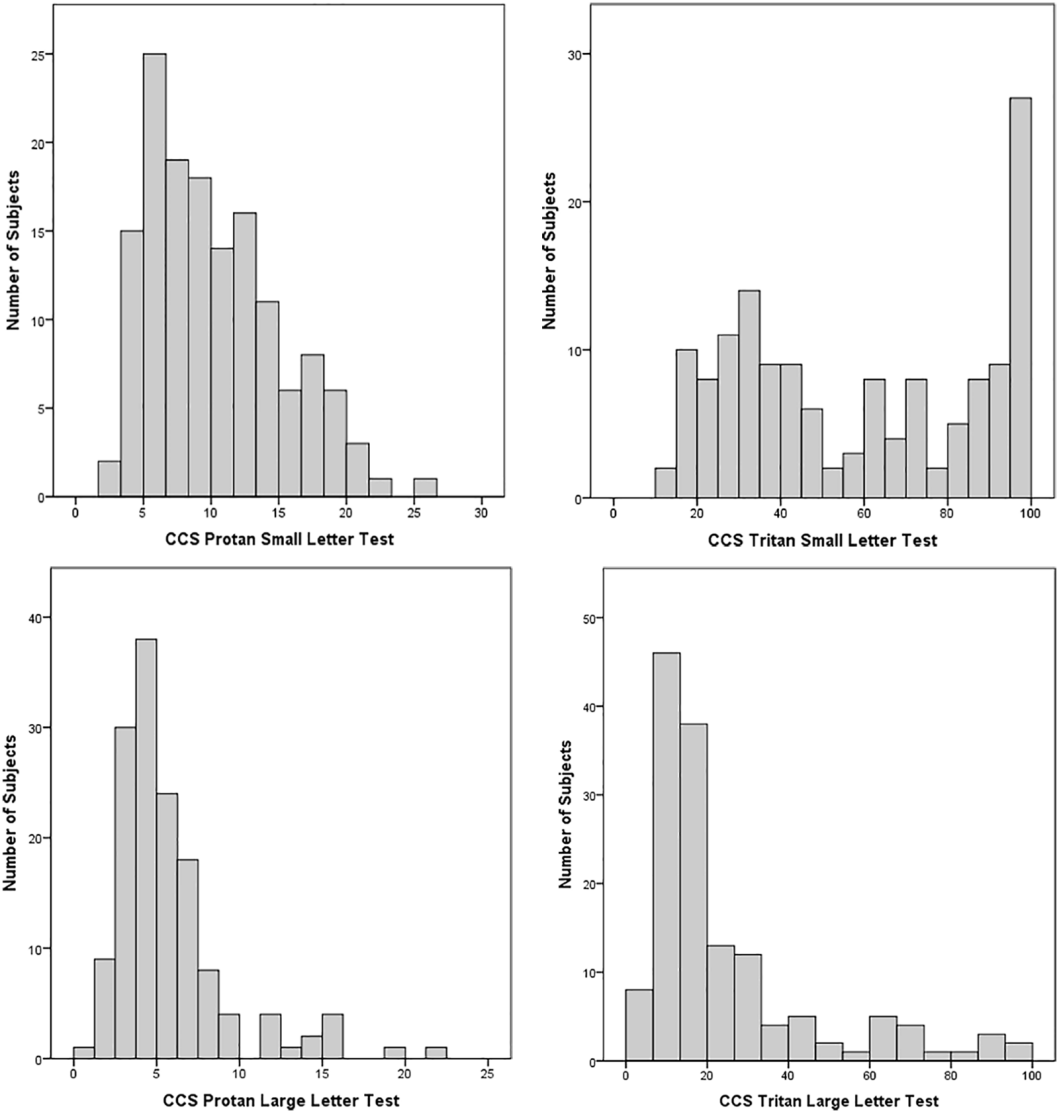
Frequency distribution of colour contrast sensitivity (CCS): higher CCS values indicate weaker colour vision.

Statistical analysis was conducted on 145 subjects (53 males, 92 females. Mean age = 75.5 years, SD = 8.3 years, range = 50–90 years). Table 1 shows a summary of results, and Figure 1 shows the frequency distribution of CCS across the four sub-tests. Table 2 shows a summary of the correlations between the variables.

**Table 1.** Normative values.

	Age	LogMAR	PS	TS	PL	TL
Mean	75.5	0.1	10.3	58.6	5.6	24.3
SD	8.3	0.1	4.8	29.4	3.5	21.5
Minimum	50	−0.1	2.0	13.4	1.2	4.8
Maximum	90	0.2	26.3	98.7	22.1	98.7

PS: protan small letter test; TS: tritan small letter test; PL: protan large letter test; TL: tritan large letter test; SD: standard deviation.

**Table 2.** Summary of correlations between variables.

		LogVA	PS	TS	PL	TL
*Age*	rho	0.14	0.32	0.35	0.35	0.40
	*p*-value	0.10	<0.005	<0.005	<0.005	<0.005
*LogVA*	rho		0.36	0.24	0.15	0.29
	*p*-value		<0.005	<0.005	0.08	<0.005
*PS*	rho			0.49	0.77	0.57
	*p*-value			<0.005	<0.005	<0.005
*TS*	rho				0.45	0.82
	*p*-value				<0.005	<0.005
*PL*	rho					0.59
	*p*-value					<0.005

PS: protan small letter test; TS: tritan small letter test; PL: protan large letter test; TL: tritan large letter test.

### CCS versus age

**Figure 2.**
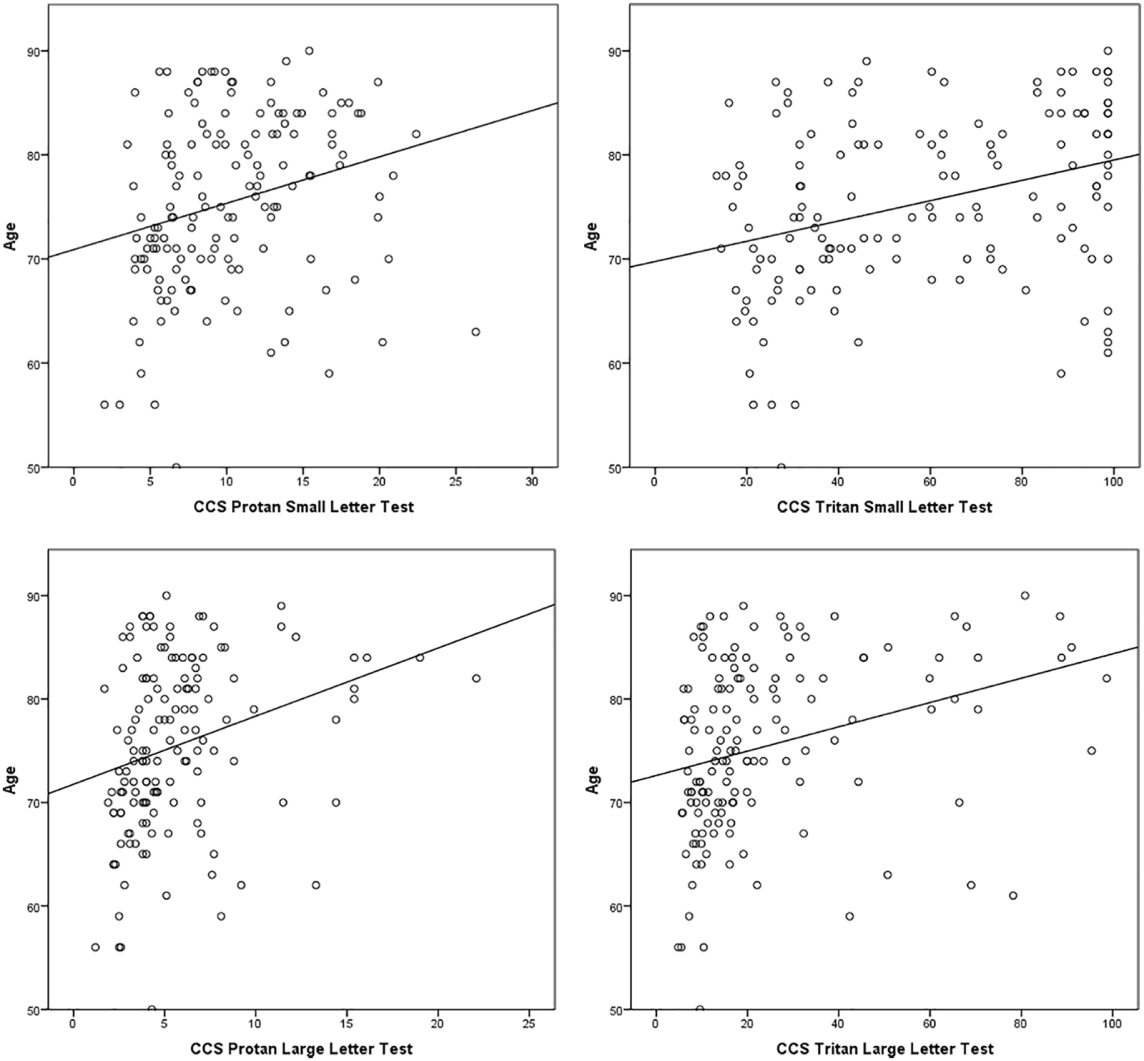
Relationship between age and colour contrast sensitivity (CCS): higher CCS values indicate weaker colour vision.

The average age in the cohort of subjects was 75.5 (range: 50–90; SD = 8.3) years. The relationship between CCS and age was investigated (Figure 2) using Spearman’s rho correlations. There was a moderate, positive correlation between the two variables for all CCS types, with higher CCS values (i.e. weaker colour vision) associated with increasing age. All correlations were statistically significant (*p* < 0.0005).

### CCS versus visual acuity

**Figure 3.**
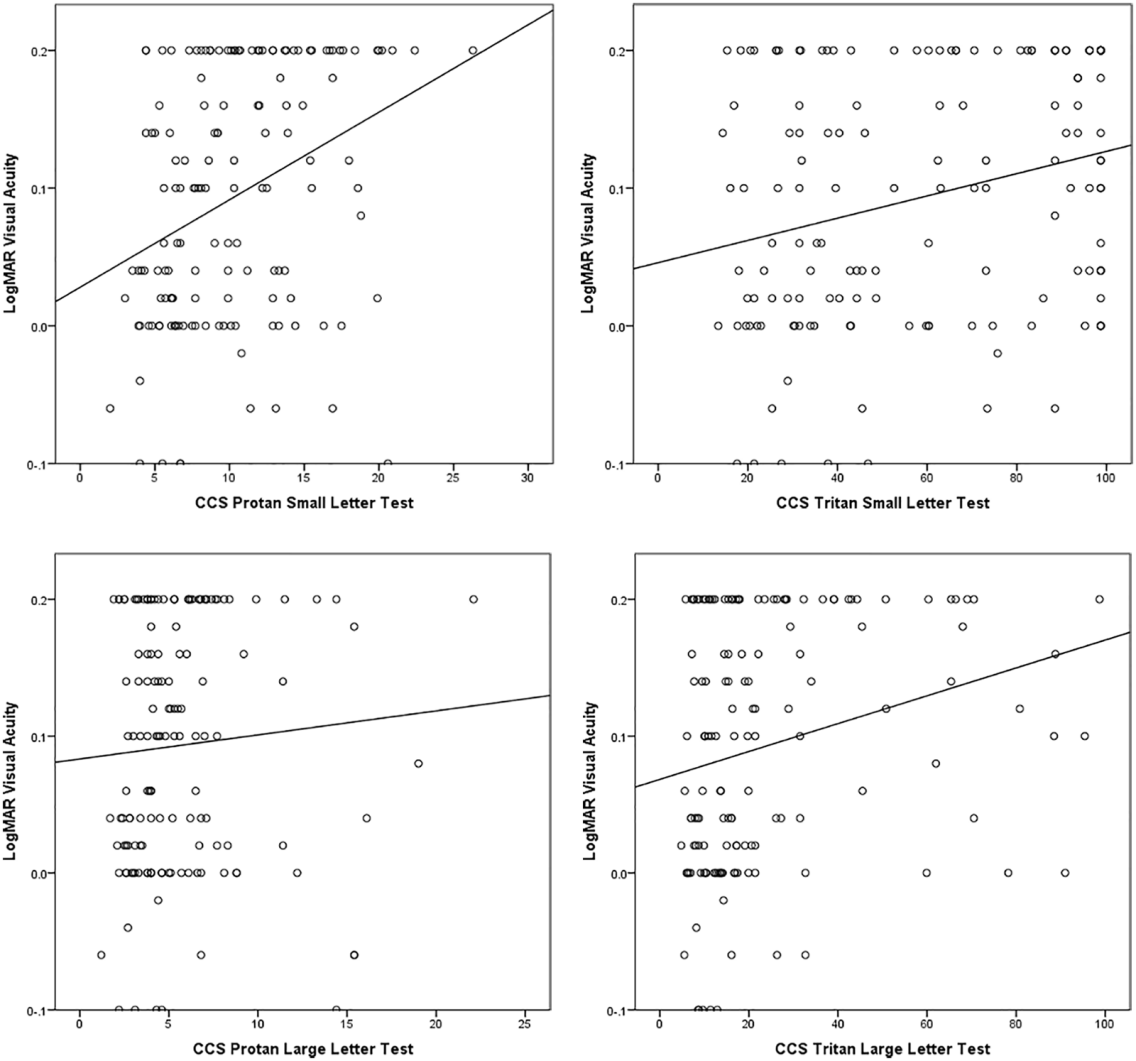
Relationship between LogMAR visual acuity and colour contrast sensitivity (CCS): higher CCS values indicate weaker colour vision.

The average LogMAR visual acuity in the tested subjects was 0.1 (range: –0.1 to 0.2, SD = 0.10). There was no significant correlation between visual acuity and age. The relationship between CCS and visual acuity was investigated (Figure 3) using Spearman’s rho correlations. Higher CCS values (i.e. weaker colour vision) were associated with poorer visual acuity, with *moderate correlation* between visual acuity and protan small letter CCS, *weak correlations* between visual acuity and tritan small and large letter CCS, and *very weak correlation* between visual acuity and the protan large letter CCS. With the exception of visual acuity versus the protan large letter CCS test, correlations were statistically significant (*p* < 0.005).

### Protan/tritan correlations

**Figure 4.**
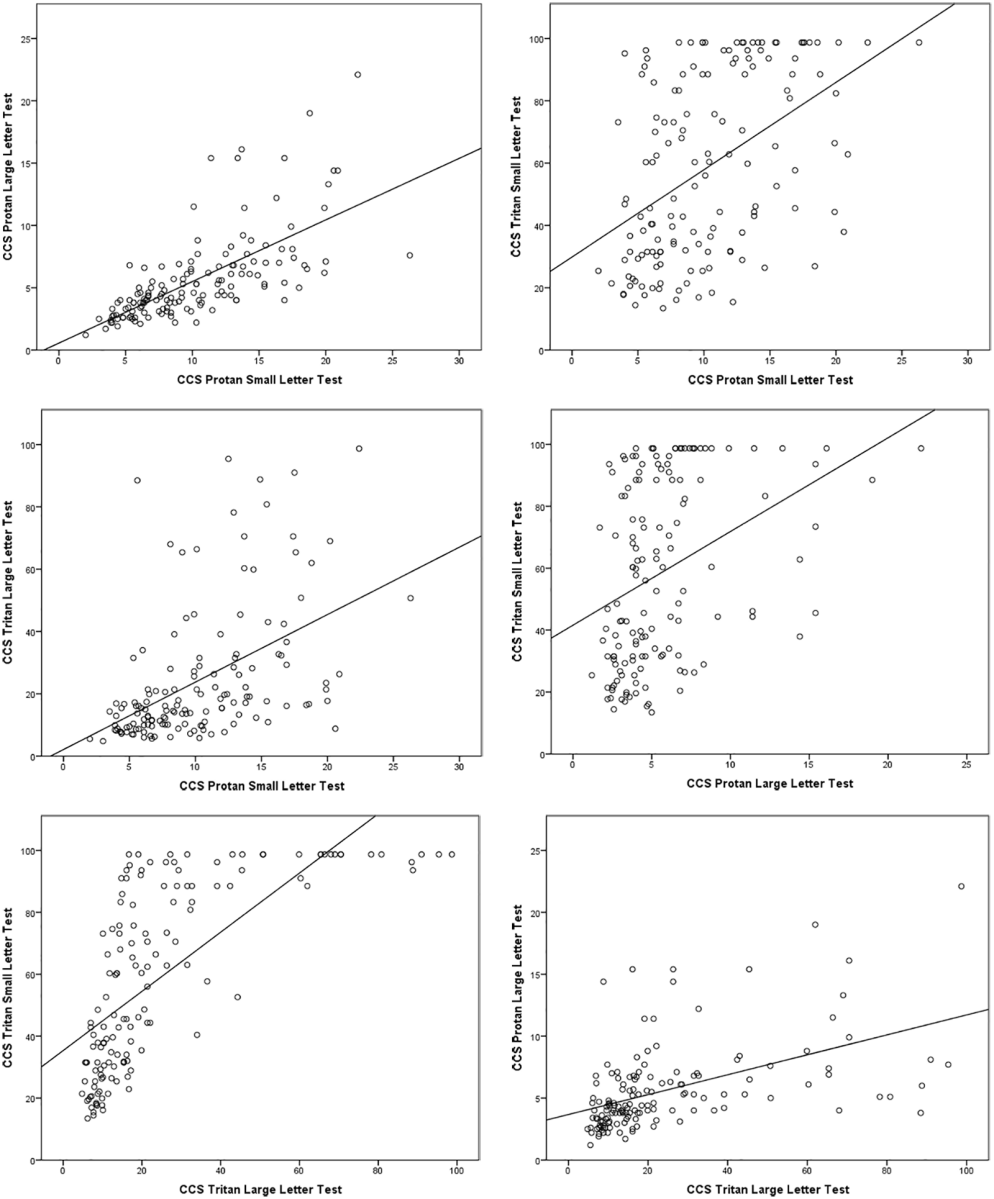
Correlation between colour contrast sensitivity (CCS) sub-tests: higher CCS values indicate weaker colour vision.

The relationship between protan and tritan CCS values was investigated using Spearman’s rho correlations. There were moderate or strong, positive, statistically significant (*p* < 0.005) correlations between all the variables, but particularly for the protan small letter versus protan large letter test and for tritan small letter versus tritan large letter test (Figure 4).

### CCS versus site, laterality and gender

There were no statistically significant differences (results not shown) in CCS levels between the three sites, between eyes (right or left) or between genders (male or female).

## Discussion

AMD is the leading cause of severe visual loss in the developed world and currently costs the UK taxpayer at least £1.6 billion a year,^4^ with the prevalence in the United Kingdom at 1.2% in individuals aged 50 years or more, 2.5% in those over 65 years and 6.3% in those above 80 years.^2^ As the population ages, AMD is likely to become a much more significant issue,^2^ with estimates suggesting the number of affected individuals is set to rise to 1.3 million by 2050.^4^ Importantly, individuals with poor vision have a higher frequency of falls and depression compared with aged-matched people with normal vision, and they are also likely to live alone and have additional health problems.^16,17^ Mild, moderate and severe sight impairment results in a 17%, 40% and 63% decrease in quality of life, respectively.^18,19^ It should be noted that a 63% decrease in quality of life is comparable to that encountered with advanced prostate cancer with uncontrollable pain, or a severe stroke that leaves a person bedridden, incontinent and requiring constant nursing care.^18,19^ Poor vision therefore significantly impacts on quality of life, and to a much greater extent than appreciated by many health professionals.

Quality of vision does not solely depend on visual acuity. Other factors, including contrast sensitivity and colour perception, are involved, and consequently, they may be relevant for quality of life, so it is important to have a means to evaluate them.

This study reveals that CCS in the fellow unaffected eye of patients with unilateral nAMD, when compared with a normative database of subjects with no evidence of macular pathology in either eye,^8^ tends to be higher (i.e. worse colour vision) than expected, especially on the tritan (blue) axis. Indeed, in many cases, especially for the small letter test, no tritan colour vision could be detected at all. This is in agreement with other studies.^8,13,14,20^ The reason CCS, especially on the yellow–blue axis, is more affected in some individuals than others is unknown; it is conceivable to postulate that sub-clinical structural damage alters the environment photoreceptors operate in, hence reducing their function. As the S-cones have a lower density in the macular area compared with the M- and L-cones, a disparity between the cone population function may be an indicator of early cell damage. This would suggest that, in some individuals at high risk of developing nAMD, macular function is partly compromised before any clinical evidence of significant, sight-threatening pathology is present and it may be possible to detect this with CCS.

The values of CCS in the unaffected eye of individuals with unilateral nAMD also show considerable variability from participant to participant throughout the cohort; this is in contrast with the relatively predictable CCS in age-matched individuals not affected by macular pathology in either eye.^8^ Again, the reason for the high inter-individual CCS variability and whether it represents a risk factor for nAMD is yet to be established.

The results of this study would suggest that in the non-affected eye of subjects with unilateral nAMD, protan and tritan CCS measured with the ChromaTest is influenced by visual acuity and age, but not gender, laterality (right or left eye) or site at which the CCS was assessed.

As would be expected, all tests being a measurement of colour vision, the four sub-tests appear to be correlated within the same individual. This holds true especially when comparing the same colour axis, that is, protan small letter test versus protan large letter test and tritan small letter test versus tritan large letter test.

Mild cataract and pseudophakia were not exclusion criteria for this study: both are more common in the elderly population and the results therefore represent the setting clinicians experience in their practice more realistically. It is known that significant lens yellowing may cause tritan deficits, due to pre-retinal absorption of short-wavelength light (in effect, a short-wavelength stop filter); future studies will evaluate the effect of mild crystalline lens yellowing on CCS in this specific cohort of patients.

## Conclusion

This study has generated a valuable database of CCS values in the fellow unaffected eye of individuals with unilateral nAMD in the clinical setting, which may serve as a reference for future projects evaluating colour vision in a cohort of subjects with the same disease entity.

Cost-effective screening for eyes at risk of developing nAMD is essential if early treatment, before irreversible damage occurs, is to be achieved, thus avoiding the significant personal suffering and socio-economic burden associated with sight loss from nAMD. With the likely rise in cases of nAMD as the population ages, the need for a cost-effective screening method becomes critical. A wealth of literature^9–12,20–24^ suggests that functional abnormalities in the retina start when the patient affected by nAMD is still asymptomatic and before currently available non-invasive imaging techniques are able to detect any abnormality. Further research is needed to establish if CCS can be utilised to identify people at imminent risk of developing nAMD and those with the early, pre-symptomatic stage of the disease, when treatment is likely to be most effective, and is the subject of ongoing work by the authors. If CCS is found to be an effective screening tool for nAMD, the equipment required is relatively cheap and non-invasive, and could be further miniaturised for convenient screening through high street optometrists. Finally, any future research should also include other tests that may act as predictors or biomarkers of nAMD, such as chromatic dark-adapted perimetry,^25^ retinotopic rod function^26^ and retinal oximetry,^27^ to name but a few, in order to establish if any can inform the clinician as to the actual risk of developing nAMD, so this highly debilitating pathology can be diagnosed and treated at an early stage.

## References

[1] Bunce C Xing W Wormald R. Causes of blind and partial sight certifications in England and Wales: April 2007–March 2008. Eye 2010; 24: 1692–1699. 20847749 10.1038/eye.2010.122

[2] Owen CG Jarrar Z Wormald R et al. The estimated prevalence and incidence of late stage age related macular degeneration in the UK. Br J Ophthalmol 2012; 96(5): 752–756. 22329913 10.1136/bjophthalmol-2011-301109 PMC3329633

[3] Klein R Cruickshanks KJ Nash SD et al. The prevalence of age-related macular degeneration and associated risk factors. Arch Ophthalmol 2010; 128: 750–758. 20547953 10.1001/archophthalmol.2010.92 PMC2896217

[4] Macular Society. Age-related macular degeneration: collaborating to find a cure. Andover: Macular Society, 2016, https://www.macularsociety.org/sites/default/files/downloads/AMD%20Collaborating%20to%20find%20a%20cure%20Accessible%20FINAL.pdf (2016, accessed 21 August 2018).

[5] Gehrs KM Anderson DH Johnson LV et al. Age-related macular degeneration: emerging pathogenetic and therapeutic concepts. Ann Med 2006; 38(7): 450–471. 17101537 10.1080/07853890600946724 PMC4853957

[6] Hammond CJ Webster AR Snieder H et al. Genetic influence on early age-related maculopathy: a twin study. Ophthalmology 2002; 109(4): 730–736. 11927430 10.1016/s0161-6420(01)01049-1

[7] Luthert PJ. Pathogenesis of age-related macular degeneration. Diagn Histopathol 2011; 17: 10–16.

[8] Arden GB Wolf JE. Colour vision testing as an aid to diagnosis and management of age related maculopathy. Br J Ophthalmol 2004; 88(9): 1180–1185. 15317712 10.1136/bjo.2003.033480 PMC1772298

[9] Kamei M Hollyfield JG. TIMP-3 in Bruch’s membrane: changes during aging and in age-related macular degeneration. Invest Ophthalmol Vis Sci 1999; 40(10): 2367–2375. 10476804

[10] Hollyfield JG. Age-related macular degeneration – the molecular link between oxidative damage, tissue-specific inflammation and outer retinal disease: the Proctor lecture. Invest Ophthalmol Vis Sci 2010; 51(3): 1275–1281. 10.1167/iovs.09-4478 20185837

[11] Falsini B Ziccardi L Stifano G et al. Temporal response properties of the macular cone system: effect of normal aging and age-related maculopathy. Invest Ophthalmol Vis Sci 2007; 48(10): 4811–4817. 17898308 10.1167/iovs.07-0306

[12] Holz FG Gross-Jendroska M Eckstein A et al. Colour contrast sensitivity in patients with age-related Bruch’s membrane changes. Ger J Ophthalmol 1995; 4(6): 336–341. 8751098

[13] Madill SA. Empirical and psychophysical correlates for the physiological processes underlying age-related macular degeneration. London: University of London, 2010.

[14] Sivagnanavel V Madill SA Arden GB et al. Correlation of colour contrast sensitivity with stage of age related macular degeneration. Invest Ophth Vis Sci 2005; 46: 1398.

[15] Gregor Z Bird AC Chisholm IH. Senile disciform macular degeneration in the second eye. Br J Ophthalmol 1977; 61(2): 141–147. 843512 10.1136/bjo.61.2.141 PMC1042899

[16] Evans JR Fletcher AE Wormald RP. Depression and anxiety in visually impaired older people. Ophthalmology 2007; 114: 283–288. 17270678 10.1016/j.ophtha.2006.10.006

[17] Legood R Scuffham P Cryer C. Are we blind to injuries in the visually impaired? a review of the literature. Inj Prev 2002; 8(2): 155–160. 12120837 10.1136/ip.8.2.155 PMC1730864

[18] Brown GC Brown MM Sharma S et al. The burden of age-related macular degeneration: a value-based medicine analysis. Trans Am Ophthalmol Soc 2005; 103: 173–184; discussion 184–186. 17057801 PMC1447589

[19] Brown MM Brown GC Stein JD et al. Age-related macular degeneration: economic burden and value-based medicine analysis. Can J Ophthalmol 2005; 40(3): 277–287. 15947797 10.1016/S0008-4182(05)80070-5

[20] Mitrut I Verma A Madill S et al. Color contrast and drusen area. Ophthalmology 2010; 117(6): 1280–1281. 10.1016/j.ophtha.2010.01.047 20522345

[21] Spraul CW Lang GE Grossniklaus HE et al. Histologic and morphometric analysis of the choroid, Bruch’s membrane, and retinal pigment epithelium in postmortem eyes with age-related macular degeneration and histologic examination of surgically excised choroidal neovascular membranes. Surv Ophthalmol 1999; 44(Suppl. 1): S10–S32. 10.1016/s0039-6257(99)00086-7 10548114

[22] Yuan X Gu X Crabb JS et al. Quantitative proteomics: comparison of the macular Bruch membrane/choroid complex from age-related macular degeneration and normal eyes. Mol Cell Proteomics 2010; 9(6): 1031–1046. 20177130 10.1074/mcp.M900523-MCP200 PMC2877969

[23] Balaskas K Nourrit V Dinsdale M et al. Differences in spectral absorption properties between active neovascular macular degeneration and mild age related maculopathy. Br J Ophthalmol 2013; 97(5): 558–560. 23137662 10.1136/bjophthalmol-2012-302305

[24] Xu H Chen M Forrester JV. Para-inflammation in the aging retina. Prog Retin Eye Res 2009; 28(5): 348–368. 19560552 10.1016/j.preteyeres.2009.06.001

[25] Pfau M Lindner M Gliem M et al. Mesopic and dark-adapted two-color fundus-controlled perimetry in patients with cuticular, reticular, and soft drusen. Eye 2018; 32(12): 1819–1830. 30068928 10.1038/s41433-018-0183-3 PMC6292882

[26] Nguyen CT Fraser RG Tan R et al. Longitudinal changes in retinotopic rod function in intermediate age-related macular degeneration. Invest Ophthalmol Vis Sci 2018; 59(4): AMD19–AMD24. 10.1167/iovs.17-23084 29860308

[27] Heitmar R Cubbidge RP. The impact of flash intensity on retinal vessel oxygen saturation measurements using dual wavelength oximetry. Invest Ophthalmol Vis Sci 2013; 54(4): 2807–2811. 23493292 10.1167/iovs.12-10493

